# An Integrated Health-System Specialty Pharmacy Model for Coordinating Transitions of Care: Specialty Medication Challenges and Specialty Pharmacist Opportunities

**DOI:** 10.3390/pharmacy7040163

**Published:** 2019-12-03

**Authors:** Autumn D. Zuckerman, Alicia Carver, Katrina Cooper, Brandon Markley, Amy Mitchell, Victoria W. Reynolds, Marci Saknini, Houston Wyatt, Tara Kelley

**Affiliations:** Specialty Pharmacy Services, Vanderbilt University Medical Center, Nashville, TN 37212, USA; alicia.b.carver@vumc.org (A.C.); katrina.cooper@vumc.org (K.C.); brandon.m.markley@vumc.org (B.M.); amy.mitchell@vumc.org (A.M.); victoria.w.reynolds@vumc.org (V.W.R.); marci.c.saknini@vumc.org (M.S.); houston.w.wyatt@vumc.org (H.W.); tara.n.kelley@vumc.org (T.K.)

**Keywords:** transitional care, pharmacy, healthcare quality, access, and evaluation, integrated delivery of healthcare, comprehensive healthcare, medication systems, programs, managed care

## Abstract

Adherence and persistence to specialty medications are necessary to achieve successful outcomes of costly therapies. The increasing use of specialty medications has exposed several unique barriers to certain specialty treatments’ continuation. Integrated specialty pharmacy teams facilitate transitions in sites of care, between different provider types, among prescribed specialty medications, and during financial coverage changes. We review obstacles encountered within these types of transitions and the role of the specialty pharmacist in overcoming these obstacles. Case examples for each type of specialty transition provide insight into the unique complexities faced by patients, and shed light on pharmacists’ vital role in patient care. This insightful and real-world experience is needed to facilitate best practices in specialty care, particularly in the growing number of health-system specialty pharmacies.

## 1. Introduction

Specialty medications have advanced the treatment of chronic and sometimes life-threatening diseases, including multiple sclerosis (MS), chronic hepatitis C viral (HCV) infection, and hematological/oncological disorders (additional disorders commonly treated with specialty medications found in Table 1). While there is no single definition of a specialty medication, common characteristics include complexity, storage, handling and delivery requirements, comprehensive patient management, manufacturer restrictions, and high cost (i.e., >$1000 per member per month [1]) [2,3]. Benefits of these therapies range from improving patients’ quality of life and productivity to prolonging life expectancy or providing a cure. Most new drug approvals (39/59) by the U.S. Food and Drug Administration (FDA) in 2018 were specialty medications [4]. The rapid rise in specialty medication utilization has introduced unique challenges to the healthcare system, most notably managing their high costs, with some costing more than $100,000 per patient per year [5,6,7]. Pharmacy benefit managers (PBMs) reported that specialty medications in the United States comprise 1–2% of prescription claims, but account for 45% of pharmacy spending, and are projected to rise to 60% of pharmacy spending in 2020 [8,9]. Healthcare providers who prescribe specialty agents struggle to navigate the complex and often time-consuming process of obtaining payer approval and financial assistance for patients to access treatment [10,11,12].

Beyond the cost of specialty medications themselves, patients with specialty conditions incur high medical costs from frequent laboratory or imaging tests to monitor medication safety and effectiveness, hospitalizations or emergency care to treat disease flares, and visits to multiple healthcare providers for comorbid conditions. Patients on specialty medications face various transitions in care, such as changes in providers or medications, or admission or discharge from healthcare facilities. Because lapses in therapy can jeopardize patient safety and treatment efficacy, ensuring adherence to therapy during these transitions is vital.

A growing number of health systems have developed internal specialty pharmacies to alleviate barriers to accessing specialty medications, improve appropriate utilization and optimization of specialty agents, and streamline transitions in care [13,14,15,16,17]. Several studies have demonstrated the benefit of pharmacists in maintaining continuity of care for patients transitioning from one healthcare setting to another [18,19]. However, little work has explored distinctive transitions in care faced by patients on specialty treatments or assessed how pharmacist involvement could ease these transitions.

The purpose of this review is to describe unique transitions in healthcare settings, healthcare providers, among specialty medications, and financial coverage for specialty medications encountered by an integrated health-system specialty pharmacy. We also discuss the specialty pharmacist’s role in guiding patients through these transitions to ensure treatment adherence and optimal patient outcomes. The descriptions of these transitions and specialty pharmacists’ actions to address the transition are based on experience from one integrated health-system specialty pharmacy established in 2012, with 23 clinical areas of involvement. The manuscript was reviewed by a different health-system specialty pharmacy who added additional insight. However, there are many models of health-system specialty pharmacy wherein the roles and responsibilities of the specialty pharmacist discussed below may vary from what is described herein or be performed by other specialty pharmacy staff members (i.e., technicians and patient advocates). The actions of the specialty pharmacist noted in this paper, therefore, refer to the specialty pharmacy team as a collective and are the experience of one large health-system specialty pharmacy. Specific case examples for each transition type are provided in Table 2. Figures illustrate transitions in care encountered by specialty patients and pharmacist interventions for each scenario.

## 2. Transitions in Sites of Care Utilized by Specialty Patients

### 2.1. Transition Considerations

Patients prescribed specialty medications are often more complex and have worse self-reported health states and more comorbidities than patients using non-specialty medications [20]. Patients with specialty diseases may have frequent disease relapses or flares resulting in urgent care visits, emergency department visits and hospitalizations. In a 2017 study exploring predictors of hospital readmissions among pediatric patients with IBD, over 20% required a repeat hospitalization within 90 days of the index admission [21]. A study of 131 patients with World Health Organization Group I pulmonary arterial hypertension found 27% of patients required hospitalization during the one year study period [22]. Patients with multiple sclerosis (MS) are at risk of relapses and infections (e.g., pneumonia), both of which are common causes of hospitalizations. With increased use of disease-modifying therapies (DMTs) (which reduce the frequency and severity of relapses), MS-associated hospitalizations in Finland have steadily declined by 4.6% from 2004 to 2014. However, frequency of infection-related admissions—which require longer length of stay and display higher in-patient mortality than relapse-associated hospitalizations, remained unchanged over the ten-year study [23].

Specialty therapy must be managed when patients are admitted to the hospital, during their inpatient stay, and at discharge or transition to another care center to ensure treatment persistence (if appropriate), prevention of drug interactions, facilitation of timely initiation of prescribed therapy, and reduction in the length of hospital stay.

### 2.2. Transition into Hospital

Upon hospital admission, it is important to determine whether a patient must continue a previously prescribed specialty medication during hospitalization. The medical necessity and urgency of administering a specialty medication to a hospitalized patient should be based on a patient’s disease state, health and disease status, reason for admission, and the risks versus benefits of continued therapy. For example, in a hospitalized patient with Crohn’s Disease, administering adalimumab for a flare may be appropriate; however, if that patient were admitted for a serious infection, adalimumab administration would not be recommended.

### 2.3. During Hospitalization

Patients sometimes initiate a new specialty therapy while hospitalized, such as anakinra for neonatal-onset multisystem inflammatory disease, and venetoclax for acute myeloid leukemia. A new specialty medication could be dispensed from the inpatient or care facility pharmacy or as a home-supplied medication. This decision requires coordination between both inpatient and outpatient care teams to ensure the patient receives his or her medication in a timely manner and is adherent to hospital or facility policy. Often, hospital administration or the Pharmacy and Therapeutics Committee must approve the use of high-cost specialty medications to hospitalized patients. Some policies also require the medication be approved through insurance for outpatient use before it can be initiated in the hospital or facility, to ensure continuity of care at discharge. Some institutions allow or require the patient to bring the medication from home if it is not stocked at that hospital or if it will not be covered by the hospital formulary; this practice, termed “brown bagging” is controversial at most institutions as the integrity of the product cannot be confirmed [24].

If a therapy is denied for inpatient administration, therapy initiation may be delayed, which could adversely affect the patient’s disease status. In patients with IBD, treatment delays may prolong the time to disease remission. Hospital formulary preferences also pose an unknown risk to the patient by requiring use of alternate therapies. For example, if a patient is initiated on infliximab while inpatient, the hospital formulary can mandate a biosimilar, such as Inflectra^®^ (infliximab), be administered to the patient for the first induction dose. Once discharged, the patient’s insurance could require the patient to switch to Remicade^®^ (infliximab). While these are both versions of infliximab, no studies have validated the efficacy of switching products during the induction phase. The interchangeability of biosimilars is still yet to be determined, which poses potential risk to patients such as lower treatment efficacy or likelihood of remission.

Monitoring and mitigating drug interactions during a hospitalization is crucial as medications are frequently added or adjusted that might interact with a specialty medication. For example, proton pump inhibitors, which are often added inpatient for stress ulcer prophylaxis, can decrease the efficacy of expensive HCV medications [25].

Additionally, some admitted patients need medication to be crushed for tube feeds or feeding issues [26]. Specialty medications are commonly available as a single formulation and careful attention must be used when altering their administration method. For example, the exposure of glecaprevir, a medication used to treat HCV, is decreased by 27–61% when crushed [27].

### 2.4. Hospital Discharge

At the time of discharge, a patient is re-evaluated to assess whether newly initiated treatments should be continued, therapies that may have been previously held should be re-initiated, and if any new potential medication interactions have emerged. Medications initiated while admitted must be obtained by an outpatient pharmacy. PBMs may require the use of a PBM-preferred specialty pharmacy, creating confusion if a patient receives all medications except the specialty medication at discharge. Additionally, it may take several days to weeks for the patient to obtain the specialty medication because a prescription must be sent to the PBM-approved specialty pharmacy, and a prior authorization may be required, before the medication is shipped to the patient.

Patients may also transition from a hospital to a skilled nursing facility (SNF). Following a hospital stay, 20% of fee-for-service Medicare beneficiaries are admitted to SNFs for post-acute care [28]. In a survey of patients transitioning from hospitals to 39 different SNFs, many patients reported they did not feel appropriately prepared for transition or educated about their post-acute needs. Many patients also noted that transitions felt chaotic because of problems with timing and medications [28]. SNFs will often refuse admission to patients receiving high cost specialty medications to avoid the liability of having to supply these medications. Some SNFs will allow patients to supply their own specialty medication, which logistically makes it difficult to determine how to get the medication to the patient. It is crucial that these details are worked out prior to hospital discharge.

### 2.5. Integrated Health-System Specialty Pharmacist Role

**Figure 1:**
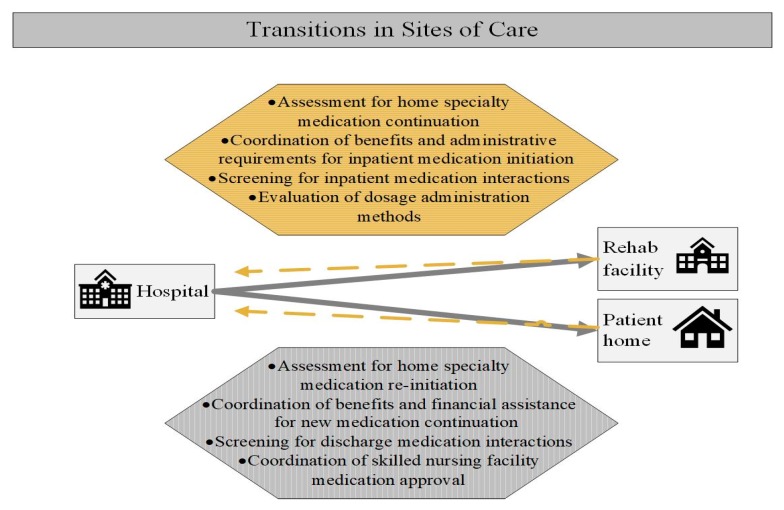
Specialty pharmacist actions during transitions in sites of care by specialty patients. (Specialty patients may transition into the hospital from community settings such as a home or rehabilitation facility, and subsequently return to these community settings at discharge. Considerations and specialty pharmacist actions during these transitions are presented. Gold dashed lines indicate transition from a community setting into a hospital. Grey solid lines indicate transition from a hospital admission into community settings. Square boxes contain sites of care; hexagons contain pharmacist actions).

Integrated specialty pharmacists play a critical role in coordinating specialty medication management during transitions between care sites (Figure 1). Because pharmacists understand potential risks of missing doses of specialty medication therapies, they can advocate for the patient and serve as a liaison between the inpatient clinical care team and the hospital administration. While the hospital team treats the acute needs of admitted patients, an integrated specialty pharmacist coordinates care to ensure ongoing access and preventing clinically relevant lapses in therapy. Effective care coordination is accomplished through frequent communication between the inpatient and outpatient healthcare teams, often through the medical record. Electronic medical records (EMRs) are a powerful tool to coordinate the various needs of a single patient throughout many care environments [29]. Documentation can include, but is not limited to, prior authorization approval information, patient’s last medication fill date, plan for accessing the medication post-discharge, and counseling provided for new medication starts. When this important information is in the patient EMR, the entire healthcare team is aware of the plan for the specialty medication therapy.

In addition to written communication, developing a collaborative relationship with the inpatient pharmacist on the patient’s care team is useful. The inpatient pharmacist can update the specialty pharmacist on any changes in the care plan and can assist with obtaining the medication, if needed, on the inpatient side. Procedures may vary by specialty, but all should evaluate the necessity of starting therapy while inpatient, determine the medication cost, determine inpatient and outpatient medication access and communicate the care plan to the patient. The outpatient specialty pharmacist can provide potential outpatient access and copay information to the inpatient pharmacist.

Finally, a standardized procedure for initiating specialty therapy during an inpatient stay can reduce the time to starting therapy and ease the concerns of the patient continuing the medication upon discharge. Coordinated inpatient and outpatient efforts are needed to ensure the patient will receive a newly-initiated medication once discharged. Ideally, a specialty pharmacist is aware of the discharge ahead of time and can preemptively address barriers to outpatient medication access to ensure no gaps in therapy and prevent costly delays in discharge.

## 3. Transitions in Provider Types seen by Specialty Patients

### 3.1. Transition Considerations

Compared to non-specialty patients, patients receiving specialty medications are more likely to require care for multiple comorbid conditions [30]. As a result, specialty patients usually receive care from multiple healthcare providers and specialists, which can pose operational challenges in managing their specialty medications. Unfortunately, most payment systems do not explicitly reimburse any provider for coordination of care efforts and there are no clear criteria for successful coordination of care, making it challenging for healthcare teams to facilitate consistent transitions [31].

Suboptimal communication between providers, patients, and other members of the healthcare team during these transitions can result in gaps in care and lapses in therapy. Patients sometimes receive inconsistent information from multiple providers regarding their specialty medication, which may cause confusion and potentiate premature discontinuation of therapy [31]. In a survey of 422 patients with chronic illness, patients who reported better primary care communication and coordination of care reported fewer “hassles” such as lack of information about medications, side effects from medications, and uncertainty about when and how to take medications [32]. Patients may report side effects to the pharmacist, non-prescribing provider’s office, or the prescriber’s office. Communication amongst these individuals is vital to ensure side effects are adequately managed and patients can continue effective therapy.

Seeing multiple providers using disparate medical record systems can also result in potentially harmful medication changes. If a non-specialty provider prescribes a new medication, the patient could be at risk of serious drug/drug interactions unknown to the provider, which could cause clinically significant changes in the safety and efficacy profile of the specialty medication. For example, initiating strong CYP3A inducers can reduce the efficacy of potentially curative hepatitis C treatment, and concomitant use of CYP3A inhibitors require dose reductions of chemotherapeutic agents due to increased toxicity.

Geographical changes may also pose challenges to managing specialty therapy. If the patient relocates or changes providers, appropriate medication monitoring may not be performed. This could result in lower effectiveness of medication due to suboptimal dosing, toxicities due to overdosing, or other compromises to patient safety, such as undiagnosed infection in patients receiving biologic agents. Additionally, patients who relocate are sometimes confused when specialty medication procurement or the medication administration site changes.

### 3.2. Integrated Health-System Specialty Pharmacist Role

**Figure 2:**
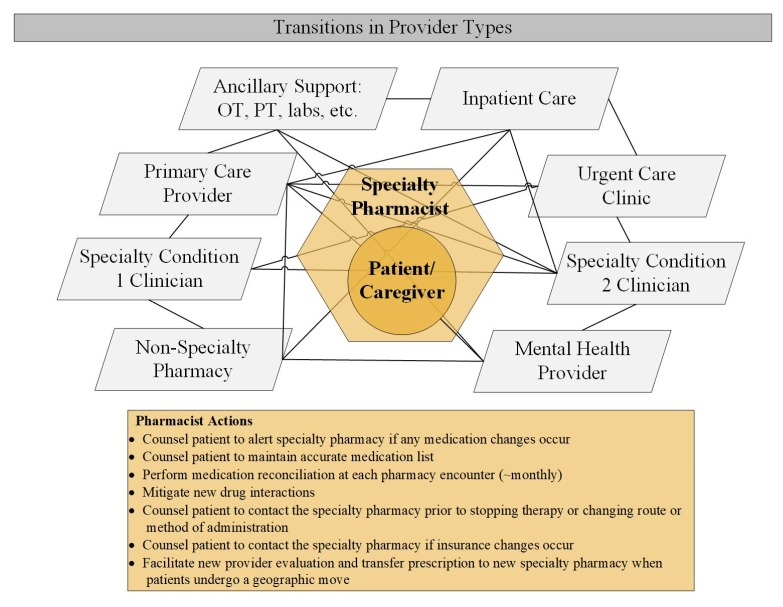
Specialty pharmacist actions during transitions in provider types by specialty patients (Patients may have multiple specialty conditions, be required to use multiple pharmacies, and see multiple providers health care professionals involved in a patient’s comprehensive care in a nonlinear or chronological manner. Lines represent movement between providers and healthcare services. Pharmacists actions in coordinating these multiple sites of care are outlined in the yellow rectangle).

Integrated specialty pharmacists in the ambulatory care setting have demonstrated improved outcomes in patient adherence and access to specialty medications [33,34,35]. To achieve these outcomes, pharmacists must oftentimes overcome the communication, side effect management, drug interaction, and geographic movement challenges noted above. Integrated specialty pharmacists are uniquely positioned to bridge the gap between providers, given their access to the EMR and their accessibility to patients (Figure 2). Pharmacists can document accurate and timely communication between providers in the EMR.

Pharmacists also conduct thorough medication education with patients initiating new medications, which is necessary for patient understanding of not only the medication, but also the process of obtaining and persisting on treatment regardless of provider or geographic transitions. New treatment education often includes detailed information regarding side effects and mitigation strategies with explicit instructions to contact either the prescribing provider or the pharmacist prior to stopping treatment, even if instructed to discontinue treatment by another provider. If the patient is instructed to discontinue treatment by another provider, the specialty pharmacist can facilitate communication between providers to ensure the appropriate recommendation for the patient. Medication reconciliation is completed during each patient contact so the specialty pharmacist can work with other providers to circumvent and resolve potential drug/drug interactions with the specialty medication.

Pharmacists can also perform chart reviews to assess if any labs are needed for medication monitoring, and to facilitate these labs being drawn when needed. Because patients can receive conflicting information from different providers, specialty pharmacists serve as a mediator to navigate and advise the patient on the best course of action.

## 4. Transitions in Specialty Medications

### 4.1. Transition Considerations

Patients often change specialty therapies due to adverse events or lack of effectiveness. In patients with hematology and oncology malignancy, disease progression or significant adverse effects may drive frequent changes to the prescribed dose, dosing administration method, and therapeutic agent. Challenges can arise when a provider makes a clinical decision to change a patient’s treatment regimen, as the new medication usually requires insurance approval, which could lead to a lapse in treatment [36]. Challenges can also occur when changing from one route of administration to another, such as transitioning from an intravenous (IV) infusion to a self-administered subcutaneous (SubQ) injection or to an oral (PO) therapy. Additionally, some oral specialty medications are given in combination with IV products. Because IV treatments are typically administered in clinic or at an off-site infusion center, regimens that necessitate both IV and oral therapies require coordination in timing of initiation and dosing of each therapy. Patients face many barriers in obtaining medication due to high out-of-pocket costs and delays in to insurance authorization [37]. This creates potential obstacles in treatment with oral and IV combination treatments or with an IV to oral treatment transition. A multiple myeloma patient needing to start bortezomib (IV)/ dexamethasone (IV)/lenalidomide (PO) (per National Comprehensive Cancer Network (NCCN) guideline recommended therapy) may start bortezomib/dexamethasone without lenalidomide if there are delays in obtaining lenalidomide [38]. Patients experiencing delays in starting a combination treatment regimen without a medication recommended by current treatment guidelines could have adverse clinical outcomes [39].

Insurance and billing challenges can also arise when changing medication, because the route of medication administration often determines whether the patient will be charged through their medical or pharmacy insurance benefit. Oral therapies and self-administered injectables are typically billed through the pharmacy benefits. IV infusions and medications given as an injection by a healthcare provider in a clinic setting are typically billed through the patients’ medical benefits. Changing routes of administration, therefore, requires a benefits investigation to determine which benefit channel is most cost-effective for the patient. Patients’ copayment amount can be determined in real-time claim results for medications adjudicated through pharmacy benefits, but usually not for medication billed through the medical benefit. This may lead to confusion regarding the patient’s financial responsibility and the potential involvement of copay assistance. Adding to this perplexity, different billing departments within the healthcare organization might be involved depending on if pharmacy or medical benefits are billed, which can lead to further confusion and frustration from the patient.

Many insurers have begun focusing on the site that care is provided as a means to reduce costs, which may impact whether a patient is able to administer treatment at home, in a hospital setting, or in an outpatient clinic. Patient may therefore be required to fill a medication through an external specialty pharmacy who then delivers the medication to the hospital for preparation and administration, termed “white bagging,” or instead, go directly to the infusion pharmacy for obtaining and being administered treatment. Many hospitals do not allow white bagging as the product integrity cannot be verified [40]. Additionally, having specialty infusion medications delivered from multiple pharmacies disrupts the standard process for receiving, procuring, and dispensing IV medication in the hospital pharmacy setting leading to potential errors. Patients may be informed in a mailed letter that they are no longer allowed to receive an infusion at a hospital where they have previously been administered treatment. This can cause confusion and the potential for missed treatment doses.

### 4.2. Integrated Health-System Specialty Pharmacist Role

**Figure 3:**
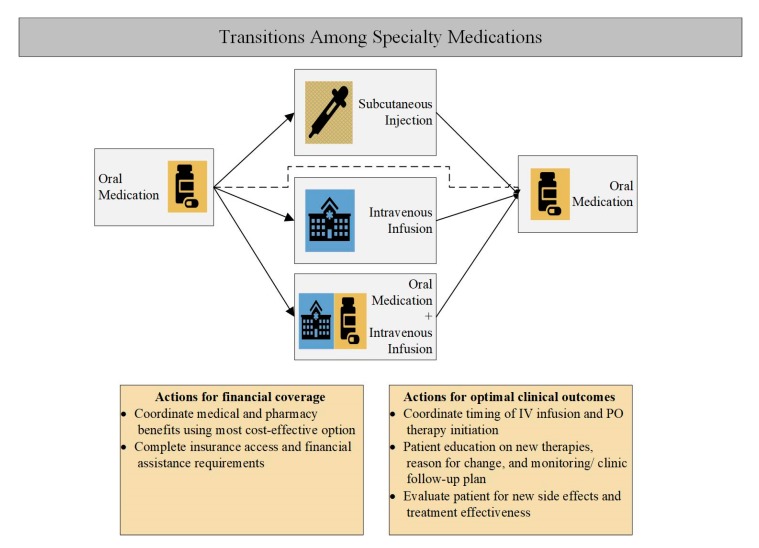
Specialty pharmacist actions during transitions in specialty medication types. (Patients with specialty conditions sometimes require the use of medications with variable administration types: oral, subcutaneous, or intravenous. Medications may be required in a subsequent manner (i.e., oral therapy, then intravenous therapy, then back to oral therapy), or be required to be administered simultaneously (oral plus intravenous therapy). The administration method of a medication may dictate which insurance coverage should be billed. In the figure blue, blue boxes indicate medications typically billed under a medical benefit; gold under a pharmacy benefit; blue and gold striped under either pharmacy or medical benefit).

Several groups are involved in securing access to a specialty therapy, including the physician’s office, patient, insurance, specialty pharmacy, and pharmaceutical manufacturer. Pharmacists can bridge gaps in care coordination between these parties (Figure 3) [41]. Navigating patient access to treatment following a change in medication administration route often requires a substantial amount of time and effort from multiple healthcare team members, which may delay treatment initiation or cause unintended gaps in therapy that can impact patients’ clinical outcomes and quality of life.

Specialty pharmacists and integrated specialty pharmacies can ensure patients’ timely transition between specialty medications and administration methods. The internal specialty pharmacy team completes all insurance authorization and financial assistance, and can communicate in real time with providers through the EMR, allowing them to ensure patients transition to new therapies without delay. Once on a self-administered therapy, pharmacists conduct follow-up calls to monitor patients for adverse events as well as missed doses and communicate concerns to healthcare providers if needed. A pharmacist’s presence in clinic can help overcome the traditional barriers to treatment with oral specialty medications.

To address confusion with sites of care related to infusion administration, some health-system specialty pharmacies have developed patient-centered programs to coordinate the process of ensuring financial coverage, obtaining the infused medication, and administering the medication in the most cost-effective manner for the patient. This alleviates patient burden, confusion, and risk of missed doses. Additionally, health-system specialty pharmacies may attempt to bill and dispense infused medications from the in-house specialty pharmacy, termed “clear bagging,” in order to ensure appropriate chain of custody, temp controls, and safety.

## 5. Transitions in Financial Coverage of Specialty Medications

### 5.1. Transition Considerations

**Figure 4:**
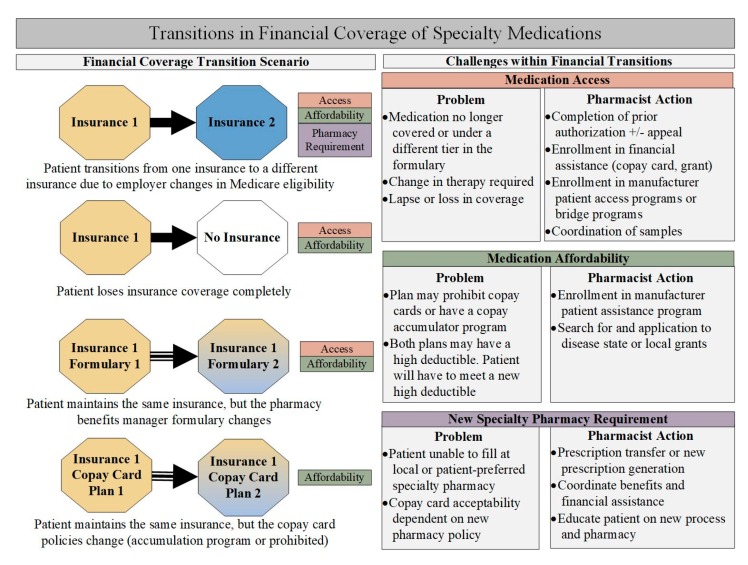
Specialty pharmacist actions during transitions in financial coverage of specialty medications. (Due to the chronic nature of most specialty conditions, patients often change financial coverage of the prescribed medication during the treatment course. Coverage changes may include transitioning to a new insurance, a loss of insurance, or changes within a single insurance plan. Octagons to the left describe scenarios of financial change with corresponding challenges resulting from the change. Boxes on the right describe problems that may arise during transitions and the action of the specialty pharmacist).

The high cost of specialty medications necessitates careful attention to consistent financial coverage of treatments to ensure adherence and persistence to therapy. Given the chronic nature of most specialty treatments, during the course of therapy patients are likely to undergo several transitions in financial coverage, including insurance changes due to employment transitions or Medicare eligibility, formulary changes within a single insurance, insurance changes in acceptance manufacturer copay cards, or loss of insurance altogether. These transitions can affect patient access to new or continued therapy, medication affordability, and the specialty pharmacy a patient must use (Figure 4).

Insurance changes may occur when a patient changes employers or loses employment. Formulary changes within a single employer may occur when the employer changes the contracted PBM or the PBM changes its formulary. Both changes would require the patient, provider, or pharmacist to seek re-approval for the specialty medication. The American Medical Association estimates that time to medication approval, on average, ranges from 24 h to 15 days [42]. In addition to a new prior authorization, if the medication is no longer preferred on the new plan and requires additional appeal information (e.g., chart notes, medical letter of necessity, and supporting scientific literature), the approval time could be delayed another 30 days. If insurance approves treatment, the out-of-pocket cost may also change if its tier in the formulary has changed or the insurance no longer accepts a manufacturer copay card. Finally, once treatment is approved and affordable, additional challenges and delays may arise if the patient is now required to use a PBM-preferred specialty pharmacy. Vertically integrated payer models that align insurers, PBMs, and specialty pharmacies continue to gain popularity as a method for managing pharmacy and medical costs [43]. The new pharmacy may not accept manufacturer copay cards, resulting in high out-of-pocket costs for the patient. These challenges may be too burdensome, and the patient may become disengaged in care and/or not obtain the medication.

Such scenarios are common and can directly impact patient outcomes. A recent study of 507 patients with relapsing remitting MS revealed nearly half of patients were currently or had previously experienced difficulty accessing a DMT, usually because of insurance prior authorization requirements and high out-of-pocket costs; more than half of patients were unable to access medication, and nearly half reported a relapse during this time. Study participants felt that delays in obtaining the prescribed DMT led to high stress, worsened their MS, and/or triggered a relapse. Several participants indicated they were forced to independently resolve DMT access issues, which often involved several weeks of dedicated time and energy [36].

Patients who reach retirement age and transition from commercial insurance to Medicare part D may incur out-of-pocket costs that are potentially thousands of dollars [44]. Patients with coverage through Medicare Part A and Part B tend to have better coverage with medications billed to the medical benefit, but financial complications still arise as the patient is not billed until after the infusion or procedure. If a patient is unaware of a change in coverage and receives a medically-billed medication that is no longer covered, they could be responsible for the entire cost of the medication. Other changes may restrict the site of care where a patient can receive their infusion or injection. If the new location is not convenient or the patient is unable to travel to the preferred location, patients may not be able to continue therapy.

Because most pharmaceutical manufacturers offer copay assistance for specialty medications, a change from one commercial insurance plan to another traditionally has yielded the same copay after the new insurance approval and copay card is applied. Recently however, there has been an increasing trend in high deductible and accumulator plans [45]. A patient who experiences a job change midyear, who had a high deductible plan with their previous employer and transitions to another high deductible plan with their new employer, may not have enough available copay card assistance from the manufacturer to make their medication affordable as funds tend to be applied on an annual basis. Accumulator plans are now prohibiting copay cards to be applied to the deductible, forcing patients to be responsible for high out of pocket deductibles before obtaining any coverage. Most patients do not have the means to afford $5000 to $10,000 high deductible plans each year and may sacrifice appropriate treatment.

Finally, patients may lose insurance altogether due to loss of employment or employer-offered benefits. Uninsured patients include patients who may be in a grace period awaiting insurance coverage from a new employer and underinsured includes patients with Medicare Part D and patients with limited specialty pharmacy coverage. Many manufacturers offer patient assistance programs that provide free drug to patients who are either uninsured or underinsured. These programs enforce maximum income requirements and may also require patients to receive treatment from a preferred pharmacy. Applications for such programs require patients to submit significant paperwork and financial documentation. Patients receiving treatment through these programs must re-apply at pre-determined intervals at which time they re-submit required paperwork.

### 5.2. Integrated Health-System Specialty Pharmacist Role

Pharmacists are gatekeepers to medication access and are uniquely positioned to recognize and offer solutions when gaps in care are identified. Patients do not always notify their physician or pharmacist about an insurance change until they are unable to obtain their medication. During initial medication counseling, it is useful for pharmacists to educate patients on the importance of alerting their pharmacy and provider’s office with each insurance change. Empowering the patient to understand the process of medication access, including insurance approval and financial assistance, from the beginning may help alleviate future delays when patients experience financial changes.

When patients incur a lapse in coverage or an insurance rejection for their medication, pharmacists and pharmacy technicians can efficiently navigate the process of ensuring medication access and affordability. Pharmacist-led PA processes have been shown to improve approval rates and significantly reduce time to PA approval [46]. High medication access rates have been reported in integrated specialty pharmacy models [33,47]. Specialty pharmacists practicing in the specialty clinic have a shared responsibility to ensure patients access and initiate treatment given their proximity to providers and patients.

Pharmacists provide solutions for patients who become uninsured or underinsured (i.e., patients with unaffordable treatment costs after insurance coverage). For temporary lapses in coverage, pharmacists will assist with enrollment in manufacturer bridge programs or coordinate medication samples or vouchers to prevent gaps in therapy. For longer lapses in coverage, pharmacists and technicians help coordinate manufacturer patient assistance program (PAP) requirements including income documentation, patient and provider signatures, and any additional required paperwork. Patients on medications without affiliated PAP programs can seek assistance through eligible disease foundation programs. Specialty pharmacists are familiar with the available programs and ways to access the assistance to decrease cost-related gaps in therapy.

Specialty pharmacists often assist in transitioning patients from an integrated specialty pharmacy to a PBM-mandated specialty pharmacy when required. The integrated specialty pharmacy must not only transfer the specialty prescription or coordinate the prescriber sending a new prescription, but also coordinate benefits and financial coverage, detailed above, as most non-health-system specialty pharmacies either do not offer these services or are less efficient in completing them. The integrated specialty pharmacist will then educate the patient about the new process of obtaining treatment from the external specialty pharmacy. These care coordination services are offered by the integrated specialty pharmacy without reimbursement and with the goal of ensuring access and persistence to needed therapies.

## 6. Conclusions

Patients taking specialty medications face unique challenges with transitions in care that may impact adherence and jeopardize the clinical benefit of these costly therapies. Former US Surgeon General, C. Everett Koop, MD, ScD once said “Drugs don’t work in patients who don’t take them” [45,48]. Specialty medications can be life changing for patients helping them to achieve low disease activity or even remission in conditions that previously had no treatment. Nonadherence caused by care transitions can lead to additional hospital visits, surgeries, medications, and even death [49]. Integrated specialty pharmacists have an opportunity to bridge gaps in care transitions by navigating medication access, promoting persistence to therapy, and coordinating communication between patients, providers, insurers, and manufacturers.

## Figures and Tables

**Table 1:** Conditions commonly treated with specialty medications.

Name
Oncology
Hematology
Multiple sclerosis
Rheumatoid arthritis
Inflammatory bowel disease
Hepatitis
Human Immunodeficiency Virus
Cystic fibrosis
Asthma
Pulmonary arterial hypertension
Bone disorders
Growth disorders
Movement disorders
Endocrinology disorders
Sickle cell disease
Idiopathic pulmonary fibrosis
Psychiatric conditions
Fertility
Nonalcoholic steatohepatitis
Hyperlipidemia
Immunology
Enzyme deficiencies
Glycogen storage diseases

**Table 2:** Example transitions of care scenarios for specialty pharmacy patients.

Specialty Condition	Example Scenario	Integrated Specialty Pharmacist Actions	Proposed Outcome
**Transitions in Sites of Care**			
Pediatric inflammatory bowel disease	Patient diagnosed with Crohn’s disease while admitted to the hospital and prescribed adalimumab	Secured approval from Pharmacy and Therapeutics Committee for inpatient administration of first induction dose Performed benefits investigation, completed access requirements for approval, and obtained financial assistance for outpatient therapy prior to treatment initiation Provided medication counseling and coordinated scheduling of teaching appointment for remaining induction dose Communicated discharge and follow-up plans to inpatient team Coordinated maintenance prescription sent to preferred specialty pharmacy	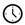 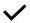 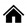 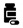
Hepatitis C	Patient started on glecaprevir/pibrentasvir inpatient following liver transplant and needed therapy to continue without interruption at discharge	Coordinated most appropriate treatment regimen with inpatient team and managed drug/drug interactions prior to starting glecaprevir/pibrentasvir Performed benefits investigation, completed access requirements for approval, including emergent appeal with peer-to-peer review, and ensured outpatient medication affordability prior to starting as an inpatient Communicated and executed discharge procurement process to patient, family, and inpatient team Coordinated clinic follow-up for safety and efficacy monitoring	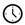 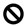 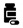 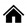
Psoriatic arthritis	Patient receiving etanercept was admitted to a rehabilitation facility that did not carry any specialty medications	Communicated medication orders and plan with physician and nursing staff at the facility Arranged delivery of the patient’s medication to be sent from the specialty pharmacy to the rehabilitation facility	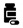
**Transition in Provider Types**			
Juvenile idiopathic arthritis	Pediatric patient receiving adalimumab moved of state	Coordinated with patient and new provider’s office to ensure continuation of adalimumab treatment Transferred the current prescription to a new pharmacy that is contracted to fill specialty medications for the patient’s insurance plan and new provider’s office Provided bridge therapy with samples until the refill could be obtained, due to the external pharmacy’s delay in filling adalimumab	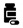
Oncology/Hematology	External provider changed antifungal prophylaxis from posaconazole to fluconazole on a patient with ongoing venetoclax therapy for acute myeloid leukemia. Patient notified pharmacist.	Communicated drug interaction with provider and prepared prescription for a dose increase of venetoclax per FDA recommendation; the provider accepted the recommendation and increased the dose	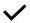 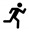
Hepatitis C	Ledipasvir/sofosbuvir prescription received from gastroenterologist for patient prescribed oxcarbazepine by psychiatrist. Significant drug/drug interaction exists between these two medications, potentially resulting in virologic failure of ledipasvir/sofosbuvir	Performed medication reconciliation at initial counseling and identified drug-drug interaction Notified gastroenterology, psychiatry, and patient of interaction Developed plan with psychiatrist to wean patient off oxcarbazepine and start lamotrigine Communicated with all parties monthly until plan completed and patient ready to start treatment with ledipasvir/sofosbuvir	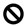
**Transitions Among Specialty Medications**			
Rheumatoid arthritis	Patient well-controlled on abatacept 750 mg IV every 4 weeks relocated to 2 h away from clinic and started to miss or be tardy for infusions	Discussed with provider and advised patient to transition to abatacept 125 mg SubQ injection weekly at home Performed benefits investigation, completed access requirements for approval, and obtained financial assistance Reviewed injection technique with patient including when to administer first subcutaneous injection based on last infusion date	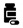 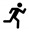 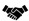
Multiple sclerosis	Patient needed to transition from natalizumab (IV infusion) to fingolimod (PO)	Performed benefits investigation, completed prior authorization and subsequent appeal Completed drug interaction and comorbidity screen to assess appropriateness of therapy Counseled patient regarding all aspects of the medication Coordinated baseline laboratory and testing requirements with patient and reviewed results to evaluate therapy appropriateness Submitted SRF to manufacturer Assisted in coordinating the FDO for fingolimod so that patient could initiate medication within 12 weeks of discontinuing natalizumab Verified patient successfully completed FDO without any significant adverse effects Sent copay assistance information and confirmed SRF to specialty pharmacy, and ensured first medication fill was delivered to patient	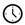 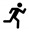
Oncology/Hematology	Patient needed to transition from bortezomib (SubQ) to ixazomib (PO)	Performed benefits investigation, completed access requirements for approval, and obtained financial assistance Counseled patient	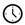
**Transitions in Financial Coverage**			
Psoriatic arthritis	Patient’s arthritis symptoms were well controlled on secukinumab, but the patient became unemployed and lost insurance and pharmacy coverage	Counseled the patient on manufacturer patient assistance program Coordinated patient and prescriber to complete required paperwork Submitted forms to manufacturer patient assistance program Coordinated medication delivery from manufacturer patient assistance program pharmacy	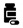 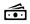
Ankylosing spondylitis	Patient was stable on golimumab 50mg SubQ monthly and received manufacturer copay card for medication. After retiring from her job, the patient transitioned from commercial insurance to Medicare, and was thus ineligible to use manufacturer copay card, resulting in an out-of-patient copayment of >$1000/month	Assessed patient for potential assistance through foundations and patient assistance programs Reviewed the patient’s new insurance and discovered she now had traditional Medicare A and B with a supplement Transitioned the patient from golimumab SubQ to IV as it would be more affordable	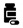 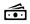
Pediatric inflammatory bowel disease	Pediatric IBD patient prescribed adalimumab. Clinic protocol was to receive first adalimumab induction dose in clinic for teaching and monitoring. Patient was unable to fill medication through the integrated specialty pharmacy due to insurance requirements.	Called insurance and received an override to fill adalimumab starter kit at the integrated specialty pharmacy so medication could be administered in clinic Scheduled induction teaching appointment with family Provided counseling and observed the first dose Monitored the patient to ensure no adverse reactions occurred Ensured maintenance dose was sent to specialty pharmacy mandated by insurer and provided information on copay assistance through manufacturer	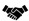 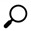
Hepatitis C	Patient diagnosed with hepatitis C was prescribed 12-week course of glecaprevir/ pibrentasvir. Medication was required to be filled through an external pharmacy. During the treatment course, patient had difficulty refilling medication due to high cost and contacted the integrated specialty pharmacist for assistance.	Contacted specialty pharmacy dispensing the medication and discovered additional financial assistance was needed for a refill of the medication to be affordable. Secured patient access to additional grant funds Communicated financial assistance with external specialty pharmacy to update the patient’s copayment amount	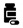

Outcomes Key: 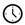 Timely treatment initiation; 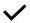 Complete or effective dosing; 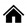 Shorter length of hospital stay; 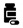 Persistence to prescribed therapy; 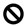 Drug-drug interaction avoided; 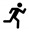 Lower risk of disease activity/progression; 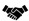 Patient education and understanding of medication administration; 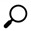 Patient monitoring; 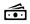 Lower medication costs. SubQ: subcutaneous; FDA: Food and Drug Administration; IV: Intravenous; PO: Oral; SRF: Start Request Form; FDO: First Dose Observation; PharmD: Pharmacist; REMS: Risk Evaluation and Mitigation Strategy; IBD: Inflammatory Bowel Disease.
